# Stress in novice nurses in new work environments: a systematic review

**DOI:** 10.3389/fpubh.2024.1463751

**Published:** 2024-10-30

**Authors:** Ángela Narbona-Gálvez, Juan Jesús García-Iglesias, Diego Ayuso-Murillo, Guadalupe Fontán-Vinagre, Juan Gómez-Salgado, Regina Allande-Cussó, Javier Fagundo-Rivera, Israel Macías-Toronjo, Carlos Ruiz-Frutos

**Affiliations:** ^1^School of Doctorate, University of Huelva, Huelva, Spain; ^2^Department of Sociology, Social Work and Public Health, Faculty of Labour Sciences, University of Huelva, Huelva, Spain; ^3^General Nursing Council of Spain, Madrid, Spain; ^4^Spanish Institute for Nursing Research, Madrid, Spain; ^5^Safety and Health Postgraduate Programme, Universidad Espíritu Santo, Guayaquil, Ecuador; ^6^Department of Nursing, Faculty of Nursing, Physiotherapy and Podiatry, University of Seville, Sevilla, Spain; ^7^Centro Universitario de Enfermería Cruz Roja, Sevilla, Spain; ^8^Department of Rehabilitation, FREMAP Huelva, Huelva, Spain

**Keywords:** nurses, occupational stress, professional burnout, psychological adaptation, mental health, clinical competence

## Abstract

**Background:**

Inexperienced nursing care can compromise the quality of care and the well-being of patients. The aim of this study was to assess the main sources of stress encountered by nurses and novice nurses in a setting not previously experienced.

**Methods:**

A systematic review was conducted following the PRISMA format in Pubmed, Scopus, Web of Science, and CINAHL electronic databases in March 2024. A total of 395 studies were identified, of which 16 met the inclusion criteria. Selection was made on the basis of topic relevance and methodological quality, assessed using the critical tools of the Joanna Briggs Institute (JBI).

**Results:**

A total of 16 studies were included in this review. Of the 16 selected, 10 were cross-sectional studies, 3 were cohort studies, 2 were qualitative, and 1 was a systematic review. The studies revealed that the main stressors for novice nurses included time management, workload, and interpersonal relationships. The results underline that organizational factors, such as lack of support and high work demands, play a key role in generating stress.

**Conclusion:**

Identifying and addressing the key challenges faced by novice nurses, such as workload, adjustment to the environment, professional expectations, and interpersonal relationships, is crucial to sustain their professional engagement and ensure the quality of health care. This understanding is essential for creating efficient policies and practices that enhance the occupational well-being and stability of nurses in the workforce.

**Systematic review registration:**

https://www.crd.york.ac.uk/prospero/display_record.php?ID=CRD42024520651, CRD42024520651.

## Introduction

The nursing profession is an essential part of the health care system, but faces significant challenges that threaten its stability, such as work-related stress, high staff turnover, and the complexity for newly graduated nurses in adapting to the workplace. These problems need to be urgently explored and addressed (1).

Stress among nursing staff is a widespread phenomenon that affects not only the physical and mental health of professionals, but also the quality of the care they provide (2). This is compounded by high expectations, overwhelming responsibilities, and limited empowerment in their daily work (3). Alarmingly, attrition rates in nursing are more than twice as high as in other health professions, which represents a significant gap (3).

The transition of newly graduated nurses from the academic environment to the world of work is a difficult process that brings mental and psychological pressures (4). Upon entering clinical practice, these professionals experience strains ranging from difficulty in communication to ethical dilemmas and overwhelming workloads (5). Lack of adequate skills and guidance contribute to increased stress and often to the onset of emotional exhaustion and burnout (4).

The COVID-19 pandemic has worsened the existing human resource shortage in the nursing profession and has increased the pressure on novice nurses, who face a more demanding and challenging work environment (6). It is crucial to understand how job stress during the first year of employment impacts on nurses’ health and on the quality of patient care. This study seeks to relate the results obtained to organizational and environmental factors that may intensify or reduce stress, with the aim of providing a deeper insight into how these factors influence the adaptation of novice nurses.

Recently, the study of job stress in nursing has gained more importance, especially through Bakker and Demerouti’s Job Demands-Resources model (7). This theory analyses how the specific demands of nursing work, such as emotional distress and its intensity, are related to the resources available to cope with these demands. Understanding this makes it possible to identify the causes of work-related stress in nursing, highlighting the importance of managing these demands and promoting resources that enhance nurses’ well-being (7). This theoretical approach will serve as a basis for exploring how the demands and available resources may influence the experience of novice nurses when entering the workplace, which is one of the main objectives of the study. In this regard, another possible problem for newly graduated nurses is the gap that may exist between the theoretical contents learnt so far at university and the reality shock that, in some cases, comes with the need to carry out a task in a short period of time (8).

The theory by Benner (9) on Nursing Competence Development highlights that as nurses gain experience and skills, they are faced with increased responsibilities, which can in turn raise their stress levels. The transition from novice to expert nurse involves taking on more complex roles and making important decisions, which adds to the emotional and psychological burden. Therefore, strategies to manage stress and provide support during their career are essential (9).

Lack of nursing experience can affect both the quality of care and the well-being of patients. Novice nurses often find it difficult to handle complex situations or to deal with patients with pathologies they are unfamiliar with or have not dealt with before, which can affect their decisions and their performance. In addition, unfamiliarity with the hospital environment and protocols generates anxiety and uncertainty, further complicating their work. It is essential to provide training and mentoring programs to support nurses as they move into more specialized roles (10).

Newly graduated nurses may experience insecurities related to their lack of experience, difficulties in integrating into work teams, and in understanding their professional role (11). These factors can make nurses more vulnerable and turn them into “second victims” after an adverse patient care event, a term coined by Wu (12) which describes the emotional distress suffered by health professionals after experiencing such a situation. It is estimated that almost half of them could go through this experience at some point in their careers, which seriously compromises their well-being and job performance (13).

The specific aim of this study is to investigate the relationship between stressors affecting novice nurses and their transitions in various work settings. In doing so, it seeks to provide a holistic view of the factors that increase vulnerability to stress, and also to identify possible interventions to facilitate a smoother transition and minimize the negative effects of stress on the quality of care provided to patients.

Overall, this study represents an analytical journey aimed at assessing the main sources of stress experienced by nurses and novice nurses in a setting new to them. The objective of this review is to provide a comprehensive and detailed overview of the challenges faced in the nursing profession by exploring different approaches and perspectives, with a view to providing valuable insights that will contribute to the formulation of more effective policies and practices that focus on the well-being of professionals and the quality of patient care.

## Methods

### Study design

A systematic review was conducted following the guidelines of the PRISMA statement (Preferred Reporting Items for Systematic reviews and Meta-Analyses) (14).

### Databases and search strategy

The search was carried out in the Pubmed, Scopus, Web of Science, and Cumulative Index to Nursing and Allied Health Literature (CINAHL) electronic databases on the basis of the key words that the research question yielded, following the CoCoPop strategy (15) (Table 1). These databases were selected on the basis of their relevance and wide recognition in the field of health sciences and Nursing, thus ensuring a comprehensive and representative review.

**Table 1:** CoCoPop format: keywords (Spain, 2024).

Condition	Stress
Context	Working in settings not previously experienced
Population	Novice nurses/nurses
Research question	*What is the prevalence of stress in novice nurses and nurses working in a new setting not previously experienced?*

The Medical Subject Headings (MeSH) thesaurus was consulted using these keywords, yielding the descriptors Disabled Persons, Barriers, and Universities. To improve the search strategy and ensure relevant studies were captured, synonymous terms were also included using Boolean operators *AND* and *OR*. Table 2 shows the search strategy applied during the search process performed on 02 March 2024 for each of the above databases. The search strategy was limited from 2014 to 2024.

**Table 2:** Search strategy used for each database (Spain, 2024).

Database	Search strategy	Results
Pubmed	(novice nurs*[Title/Abstract] OR newl* graduate nurs*[Title/Abstract] OR junior nurs*[Title/Abstract] OR newl* nurs*[Title/Abstract]) AND (Psychological Stress[Title/Abstract] OR Life Stress[Title/Abstract] OR Psychological Distress[Title/Abstract] OR Emotional Distress[Title/Abstract] OR Emotional Stress[Title/Abstract] OR Job stress[Title/Abstract] OR Occupational Stress[Title/Abstract] OR Work-related Stress[Title/Abstract] OR Workplace Stress[Title/Abstract] OR Professional Stress[Title/Abstract] OR Job-related Stress[Title/Abstract]) Filters: from 2014–2024	109
Scopus	(TITLE-ABS-KEY (“novice nurs*” OR “new* graduated nurs*” OR “junior nurs*” OR “new nurs*”) AND TITLE-ABS-KEY (“Psychologic* Stress*” OR “Life Stress*” OR “Psychological Distress” OR “Emotional Distress” OR “Emotional Stress*” OR “Job stress*” OR “Occupational Stress*” OR “Work-related Stress*” OR “Workplace Stress*” OR “Professional Stress*” OR “Job-related Stress*”)) AND PUBYEAR >2013 AND PUBYEAR <2025	101
Web Of Science	“novice nurs*” OR “new* graduated nurs*” OR “junior nurs*” OR “new nurs*” (Topic) AND “Psychologic* Stress*” OR “Life Stress*” OR “Psychological Distress” OR “Emotional Distress” OR “Emotional Stress*” OR “Job stress*” OR “Occupational Stress*” OR “Work-related Stress*” OR “Workplace Stress*” OR “Professional Stress*” OR “Job-related Stress*” (Topic) and 2024 or 2023 or 2022 or 2021 or 2020 or 2019 or 2018 or 2017 or 2016 or 2015 or 2014 (Publication Years)	136
CINAHL	AB (“Psychologic* Stress*” OR “Life Stress*” OR “Psychological Distress” OR “Emotional Distress” OR “Emotional Stress*” OR “Job stress*” OR “Occupational Stress*” OR “Work-related Stress*” OR “Workplace Stress*” OR “Professional Stress*” OR “Job-related Stress*”) AND AB (“novice nurs*” OR “new* graduated nurs*” OR “junior nurs*” OR “new nurs*”). Filters: from 2014 to 2024	49
Total		395

### Selection criteria

The following criteria were used to select the articles:

Inclusion criteria:

1. Language: Only original articles published in English, Spanish, French, or Portuguese.
2. Type: original articles, meta-analysis, systematic reviews, short communication, and case reports.
3. Population: nurses and novice nurses facing situations not previously experienced, considering novice nurses as those with less than 1 year of nursing work experience.
4. Articles measuring any of the following values and/or effects:

- Stress level of nurses and novice nurses in situations they have not experienced before.
- Stressors inherent to the nursing practice.

Exclusion criteria:

1. Studies that were classified as being of low scientific-technical quality after application of the quality assessment tool were excluded. This assessment was carried out by two reviewers independently, both of whom determined the methodological quality of the selected studies.
2. Articles that did not directly address the research question and those that were not related to the objectives of the review were excluded, ensuring that all included studies made a meaningful contribution to understanding the stress of novice nurses in new work settings.
3. Certain types of publications, such as opinion articles, editorials, and letters to the editor were excluded, as these types of articles do not provide empirical data that conforms to the objectives of this review.

### Data collection and extraction

Two researchers conducted independent searches, eliminated duplicate studies, and selected articles for inclusion based on previously established criteria after reading the abstract and title. Subsequently, the full text of potentially eligible studies was reviewed by the same two authors, and decisions to include or exclude studies were made by consensus. Any discrepancies were resolved by a third author.

### Methodological quality assessment

The methodological quality of the selected studies was independently determined by two reviewers by using the Critical Appraisal Tools for Trials of the Joanna Briggs Institute (JBI) at the University of Adelaide (16). These tools enable the assessment of a study’s methodological quality and the identification of any potential bias in its design, conduct, and/or analysis. To ensure consistency and reliability in the assessment, the reviewers met before starting the process to discuss and unify criteria. The versions for quantitative cross-sectional studies (8 items), qualitative studies (10 items), systematic reviews (11 items), and cohort studies (11 items) were used, with a cut-off point of 6 for the first two and 8 for the last two to accept their inclusion in this review. This evaluation process is detailed in the Supplementary material, which also includes a table summarizing the quality scores for each included study.

## Results

**Figure 1:**
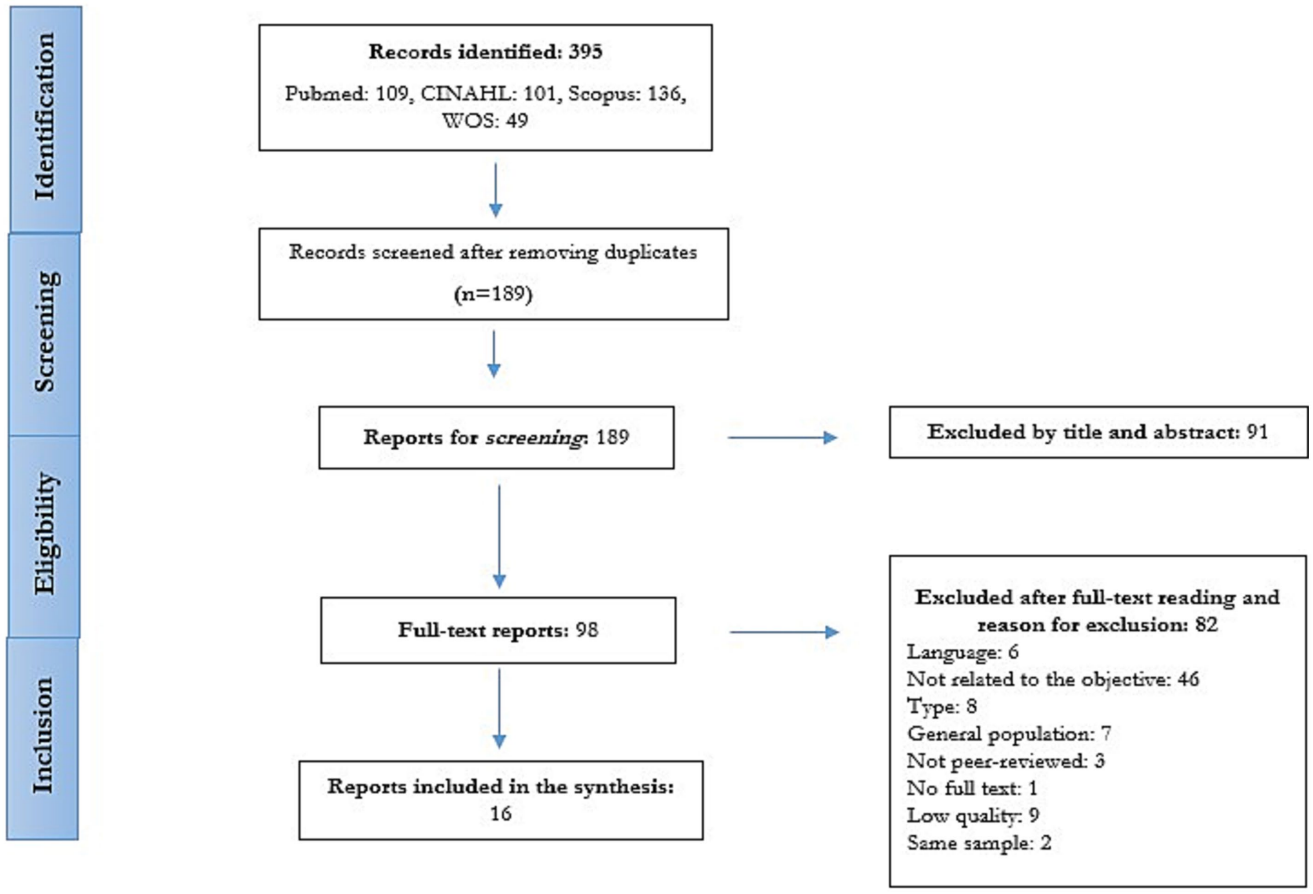
Search results (Spain, 2024).

A total of 395 references were identified by applying the initial search strategies, which were then screened according to the topic of this review. Finally, 16 studies were selected (Figure 1), of which 10 were descriptive cross-sectional studies, 3 were cohort studies, 2 were qualitative studies, and 1 was a systematic review.

Four articles were found to have been conducted in China (1, 3, 17, 18), 2 in Taiwan (6, 19), 1 in the UK (2), 1 in the Netherlands (20), 1 in Oman (4), 1 in the United States (5), 1 in South Korea (21), 1 in Brazil (13), 2 in Canada (22, 23), 1 in Denmark (24), and 1 in Jordania (25).

In 10 of the 16 selected studies (1, 3, 5, 6, 13, 17, 19–21, 24), the sample consisted of newly graduated nurses, while in 1 of the selected studies (17) the sample consisted of nurses with 1 year of experience. In another selected article2 the sample consisted of novice nurses at the beginning of their career, at 6 months, and at 12 months. In another study, the sample consisted of nurses with less than 2 years of experience (22), and another study involved nurses with less than 3 years’ experience (23). The last article to be included (4) was an integrative review including 21 review articles.

Among the interventions, the NSS scale was used (1–3), and also the PSS (17, 18) in some of the studies, while self-structured questionnaires were utilized in other investigations (3, 6, 13, 19, 21, 25). Another study used both the PSS questionnaire and the CD-RISC-25 (25). The NWI-R (23) and CERQual (24) questionnaires were also used in other articles.

The JBI critical appraisal tool was used to assess the included studies, which scored medium-high for both cross-sectional observational studies and qualitative studies.

Table 3 shows the characteristics of each of the 16 studies included in this review. The studies were classified by their authors, year of publication, country, design and objective, participants, instrument, and main results. Additionally, the results of the JBI critical appraisal tool were included.

**Table 3:** Characteristics of the studies included in the systematic review (Spain, 2024).

Studies	Context	Study objective	Type of study	Participants	Methods	Main findings	Quality of studies
Fang et al. (3)	Fujian (China)	To analyze changes in occupational stress in new nurses during the first year of employment	Analytical study	Recruited new nurses (*n* = 127)	NSS Chinese version	The results showed that new nurses had moderate to high levels of stress in all four stages, with the highest stress level at 4 and 8 months of employment and the lowest stress level at 12 months; the differences in stress scores at different time points were statistically significant (). The trends in each stressor dimension varied across different periods. The highest scores were for pressure caused by “time allocation and workload,” which peaked in month 8. The same trend was observed for stress from “patient care” and “work environment and equipment.” “Management and interpersonal relationships” scored the highest overall stress score at the start of employment before declining. The lowest stress score was from “work environment and equipment” at the start of employment, and the lowest was from “management and interpersonal relationships” from month 4 onward	6/8
Halpin et al. (2)	London (United Kingdom)	To investigate transition in newly qualified nurses through an exploration of their stressors and stress experiences during their first 12 months post-qualifying	Cohort study	Newly graduated nurses: at the time of qualification (*n* = 288), 6 months later (*n* = 107) and 12 months later (*n* = 86)	NSS	Workload was consistently the highest reported stressor with inadequate staffing and managing multiple role-demands given as explanations. Incivility within the workplace was a noted stressor. Conversely, being part of “a good team” provided a civil, supportive, facilitative work environment. Entering nurse education with previous healthcare experience had a mediating effect on the reported frequency of stressors	8/11
Hoeve et al. (20)	Groningen (The Netherlands)	Getting insight in the most crucial organizational job stressors for novice nurses’ professional commitment and whether the job stressors are mediated through negative emotions	Cohort study	Novice nurses (*n* = 18)	Semi-structured electronic diary that assesses: Organizational job stressors Emotions Work commitment: measured using the scale (RECS-E)	Path modeling revealed that lack of support from colleagues, negative experiences with patients and confrontations with existential events were most strongly negatively related to professional commitment through negative emotions. Other indirectly and negatively related organizational job stressors to commitment were complexity of care, lack of control and work-life imbalance; only conflicting job demands, and lack of control related to professional commitment directly	8/11
Labrague and McEnroe-Petitte (4)	Mascate (Oman)	To appraise and synthesize evidence relating to new nurses’ stress experiences during the transition period	Systematic review	21 review articles	PRISMA	New nurses perceived low to moderate levels of stress mainly from heavy workloads and lack of professional nursing competence. Individual and organizational factors that might contribute to their stress experiences were rarely explored	9/11
Zhang et al. (1)	Shanghai (China)	To determine differences in occupational stress levels of newly graduated nurses at different time points during the first 3 years of practice; to identify potential subgroups of nurses who perceive different occupational stress levels over time; and to evaluate differences in identified subgroups based on demographic and work-related characteristics	Analytical study	Newly graduated nurses with no experience (*n* = 152)	NSS	The entire sample of newly graduated nurses experienced a significant decrease in occupational stress during the first 3 years. The best-fitting group-based trajectory model described three distinctive trajectories: low occupational stress (19.1% of sample); medium occupational stress (67.1%) and high occupational stress (13.8%). The low occupational stress subgroup had a higher proportion of nurses from Shanghai, and the majority were employed as contact-based nurses. In comparison, the high occupational stress subgroup had the largest proportion of nurses from other provinces (outside of Shanghai), almost half of participants were employed as “bianzhi” nurses, and the majority reported to be assigned preceptor by shift	6/8
Feeg et al. (5)	New York (United States)	To understand the experiences of new nursing graduates in their work environment and the perceived stressors during their transition to the role of a competent nurse	Qualitative study	Newly graduated nurses (*n* = 1,456)	NSNA survey	Respondents felt that a high nurse/patient ratio was not safe for the patients under their care. Respondents also felt that they could not provide complete care to their patients. Respondents felt that there was not enough time to complete patient care After the first categorical analysis, the researchers elaborated on how individual items revealed meaningful statements or how new graduates were trying to find “balance” in their work, their expectations, and their interactions with others. The topics that emerged related to respondents’ overall “balance,” reflecting environmental stress (“My workload is higher than I expected”); expectations about oneself (“I do not know everything I need to know”); and interactions with others that affected them (“My relationships with others are difficult”)	9/10
Zhu et al. (18)	Shanghai, Hong Kong and Taipei (China)	to explore and compare stress, coping, professional identity and work locus of control of new graduate nurses among Shanghai, Hong Kong and Taipei.	Analytical study	Nurses with 1-year experience in the hospital (*n* = 591)	PSS Questionnaire Chinese trait coping style questionnaire Nurse professional identity scale Work locus of control scale (Chinese version)	The newly graduated nurses in Shanghai had significantly lower (*p* < 0.05) work stress score (2.65 ± 0.67) compared with their counterparts in Hong Kong (2.99 ± 0.69) and Taipei (2.94 ± 0.60). Newly graduated nurses in Shanghai tended to choose positive coping to deal with stressful situations, whereas those in Hong Kong would be more likely to adopt negative attitudes (*p* < 0.05). The newly graduated nurses in Taipei had the lowest level of professional identity (3.25 ± 0.55, *p* < 0.05), and their work control tended to be external (46.13 ± 6.20). In contrast, those in Shanghai (52.75 ± 6.04) and Hong Kong (59.41 ± 7.29) tended to be controlled internally	6/8
An et al. (21)	Gwangju (South Korea)	To examine turnover intention levels and identify the factors affecting turnover intention of new Generation Z nurses, focusing on job stress and sleep disturbance	Analytical study	New nurses (*n* = 133)	Structured questionnaire including data on job stress, sleep disturbance, and turnover intention	Most nurses were women (91.7%) and approximately two-thirds worked in the surgical ward (*n* = 61, 45.9%). Turnover intention was 12.8%, average job stress was 40.11 ± 90.7, and average sleep disturbance was 42.39 ± 15.27. New graduate nurses’ turnover intention was associated with job stress (OR = 1.07, 95% CI = 1.02–1.12) and sleep disturbance (OR = 1.19, 95% CI = 1.05–1.35), and this model explained 47.7% of the variance. Study findings determine that job stress and sleep disturbance were significant predictors of turnover intention in new nurses at the eighth week after joining the hospital	8/8
Zhou et al. (17)	Jiangsu, Zhenjiang (China)	To investigate some possible job stress factors that could influence newly recruited nurses’ behavior to either continue or discontinue their job with their organization	Analytical study	Novice nurses in 20 Chinese hospitals (*n* = 564)	PSS Questionnaire.	The effects of six job stressors from the perceived stress scale were estimated. The results showed that four stressors, stress from taking care of patients (β = 0.111, *p* < 0.01), stress from roles and workload (*β* = 0.129, *p* < 0.001), stress from co-workers and daily life (β = 0.323, *p* < 0.001) and stress from lack of professional knowledge and skills (β = 0.137, *p* < 0.001), from the perceived stress scale had a significant impact on turnover intention among nurses	8/8
Chen et al. (19)	Keelung (Taiwan)	To develop an Objective Structured Clinical Exam (OSCE) to evaluate novice nursing practitioners’ clinical competency, work stress, professional confidence, and career satisfaction	Quasi-experimental study	Novice nurses (*n* = 55)	Pre-post questionnaire (modified from a Nursing Competency Questionnaire, a Stress scale, and Satisfaction with Learning scale)	Among the novice nursing practitioners, 41 of them (74.5%) passed the exam with a mean score of 61.38 ± 8.34. There was a significantly higher passing rate among nurses who were working in medical-surgical wards (85.7%) and the intensive care unit-emergency department (77.8%) compared to novice nursing practitioners working in other units. All the novice nursing practitioners at Station A had poor performance in assessing patients with a fever. OSCE performance was more associated with educational attainment and work unit, rather than the gender. Finally, the participants showed statistically significant increases in their clinical competency, confidence in their professional competence, satisfaction with the clinical practice, and decreased work stress after the OSCE	9/9
Chen et al. (6)	Chang Gung, Chiayi (Taiwan)	To explore registered nurses’ competence in nursing care and their intention to stay in their current workplace	Analytical study	Novice nurses (*n* = 333)	Structured questionnaire to assess factors related to nursing competence based on the essentials of baccalaureate education for professional nursing practice	The results of the present study revealed that the majority of the novice nurses’ workplaces varied significantly among the transition shock. The novice nurses had the competence to provide quality care related to patient ethics, end-of-life care, and risk prevention. However, the novice nurses had the lowest average score in “utilization of care models, implementation, and leadership in the team,” indicating a lack of adequate practicum experience in leading care teams. The results indicated that the novice nurses who took infection care related courses in baccalaureate nursing programs were more familiar with relevant concepts and had greater awareness of care related concepts. The results indicated that the participants were willing to stay in their current nursing workplace and that care competence was positively correlated with intention to stay. Intention to stay was negatively correlated with clinical stress. The stepwise regression analysis revealed the following significant predictors of intention to stay: clinical stress, frequency of caring for infectious patients in the workplace, receiving nursing courses in the work field, and the frequency of care for patients in professional nursing careers	7/8
Alevi et al. (13)	São Paulo (Brazil)	To describe the prevalence of newly graduated nurses as second victims of adverse events and to know the conditions of support received in health institutions	Analytical Study	Nurses (*n* = 138)	Online questionnaire on the characteristics of the participants, the institution, and adverse events	A total of 240 responses were obtained, of which 138 were considered for analysis. Most of the participants were women, recent nursing graduates. A significant percentage had worked as nursing assistants or technicians before graduating. Many were studying in specialization or professional residency. Most of them had no institutional onboarding training when hired and did not know the term “second victim.” Around 27% had been involved in adverse events, mainly related to medication. After these events, the majority reported negative feelings and insecurity, but only 59.5% felt they had received support. Some participants had received punishment. No significant differences were found between those who had received feedback on their training and recruitment period and those who had not, in terms of being involved in adverse events	6/8
Dames (22)	Canada	To analyse how life experiences and university education influence newly graduated nurses’ ability to manage stress, maintain emotional congruence, and thrive in their work, to reduce the risk of burnout and achieve career success	Qualitative study	Female nurses under the age of 40, with less than 2 years of work experience, in British Columbia, Canada (*n* = 8)	Three 60-90-min interviews with each participant. The first two interviews were conducted 1–2 weeks apart, and the third interview, 3–4 weeks later	The most influential themes that emerged from the undergraduate factors, which interplay to impact congruence and one’s ability to thrive in their novice nurse role were frequent self-care, receiving practicum and job placement advice that promoted congruence and identity formation, curriculum components that felt personally and professionally relevant. The importance of setting authentic goals, the ability to act as change agents and the development of self-compassion were also highlighted. However, self-care was perceived as a symbolic rather than a practical concept, and some participants felt pressure to follow career paths that did not align with their preferences, leading to stress and dissonance. Emotional management and personal-professional congruence emerged as key elements in mitigating burnout	8/10
Laschinger et al. (23)	Canada	To investigate factors influencing new graduate nurses’ successful transition to their full professional role in Canadian hospital settings and to determine predictors of job and career satisfaction and turnover intentions over a one-year time period in their early employment	Cohort study	Newly graduated nurses in Canada with less than 3 years of experience (*n* = 406)	NWI-R	Authentic leadership, perceptions of structural empowerment, and person-job fit were moderate at both time points, decreasing significantly over time. At both time points, new graduates felt that their work environment was supporting, maintaining high rates of job and career satisfaction, as well as low turnover intentions. Significant correlations were found between person-job fit and psychological capital (Psycap) with job satisfaction. In addition, cynicism and Psycap were identified as significant predictors of job satisfaction and turnover intentions	7/11
Al Hadid et al. (25)	Salt (Jordania)	To examine the impact of caring for patients with COVID-19 on career decisions, resilience, and perceived self-efficacy among newly hired nurses in Jordan. It also tested whether stress, resilience, and other factors would predict intentions to stay among new nurses who care for patients with COVID-19	Analytical study	Newly hired nurses (*n* = 300)	Demographic characteristics PSS CD-RISC-25	most newly hired nurses reported low to moderate levels of occupational stress, and only around half of them showed low to moderate resilience. In addition, the majority picked nursing as their preferred job, expressing a high level of autonomy in decision-making as well as existing work conditions that were unfavorable and as a result affected their decision to continue in nursing. Only financial return was a significant predictor to stay in nursing among nurses in this study. Examining the influence of caring for COVID-19 patients on newly hired nurses’ career decisions is suggested as well as the development of interventional programs to improve their well-being	7/8
Kaldal et al. (24)	Aalborg (Denmark)	To summarize existing research syntheses reporting newly graduated registered nurses’ experiences of providing direct care in hospital settings, in order to develop evidence-based recommendations for clinical practice and research	Analytical study	44 review articles	CERQual	Newly graduated registered nurses face multiple challenges in the transition from student nurse to practicing nurse. Many experience a marked lack of competence in areas such as communication with patients and colleagues, leading to insecurity and stress. They also suffer from emotional distress and low self-confidence, which affects their ability to provide adequate care. The need for support is crucial to their professional development, as they rely on colleagues and mentors to acquire skills and manage stress. However, the work culture can negatively influence their experience, with judgmental behaviors limiting their willingness to seek help. The review emphasizes the urgency of implementing educational and organizational strategies that address these needs, as well as the importance of considering the professional identity of these nurses, as this impacts on the quality of patient care and on their job satisfaction	6/8

NSS, The Nursing Stress Scale; RECS-E, Repeated Exploration and Commitment Scale in the domain of Education; PRISMA, Preferred Reporting Items for Systematic Reviews and Meta-Analyses; NSNA, National Student Nurses’ Association; PSS, Perceived Stress Scale; OSCE, Objective Structured Clinical Examination; NWI-R, Nursing Work Index – Revised; CD-RISC-25, The Connor-Davidson Resilence Scale.

## Discussion

In various studies, such as the one by Fang et al. (3), multiple stages in which new nurses experience varying levels of work-related stress have been identified. Prominent factors include time management, workload, and interpersonal relationships. These findings are consistent with previous research, such as the one conducted by Feeg et al. (5), which emphasizes the significance of time management and adaptation to the work environment as sources of stress. The analysis highlights the inherent complexity of the nursing work environment, where time management, management skills, and interpersonal relationships play a crucial role in emotional well-being and professional performance (5). This approach is consistent with the stress model proposed by Lazarus and Folkman (26), which suggests that the way people view work demands and their ability to cope with those demands are key to understanding how they deal with stress (26). Therefore, understanding these factors is essential in order to develop effective support and training strategies to help new nurses face and overcome challenges throughout their careers (5).

Feeg et al. (5) and Chen et al. (19) address the stress derived from professional expectations among newly graduated nurses, highlighting the feeling of “not knowing everything they should know” as a significant source of stress. This pressure reflects the challenging transition from formal education to clinical practice, with resulting insecurities and anxieties. According to Benner’s transition model (1984) (9), novice nurses go through different phases in their professional development, and their sense of lack of competence is greatest in the early stages. This has been supported by recent research highlighting the importance of adequate training (9). Lack of experience, identified by Halpin et al. (2), also contributes to initial stress. However, the research by Chen et al. (19) showed that stress levels decrease with training and experience, highlighting the significance of continuous training and professional development to mitigate stress and enhance adaptation to work challenges for newly graduated nurses.

The research by Halpin et al. (2) highlighted lack of experience as a stressor in nurses, emphasizing the importance of previous experience in health care in adapting to work stress. Experienced professionals show greater confidence and effectiveness in difficult situations, which reduces their vulnerability to stress. These findings emphasize the importance of experience and ongoing education in reducing work-related stress among nurses. They also highlight the need to manage professional expectations appropriately to promote well-being and effectiveness in the clinical setting.

Hoeve et al. (20) and Zhou et al. (17) explored different stressors in the nursing work environment. Hoeve et al. (20), on the one hand, highlighted the importance of sharing experiences among newly graduated nurses and belonging to a collaborative team to reduce stress (17). The social support theory by Uchino (27) backs up this idea, indicating that support networks can help reduce the impact of work-related stress on the mental health of professionals. Underpinning this idea, Uchino’s social support theory indicates that support networks can mitigate the impact of work-related stress on the mental health of professionals (27). These findings underline the key role of peer social support as a protective factor against work-related stress. Additionally, Zhou et al. (17) assessed work-related stressors, such as caregiving stress, workload, and peer stress. To support stress management, it is crucial to understand the interpersonal dynamics in the nursing work environment and how they affect the psychological well-being of professionals. A work environment that fosters open communication and mutual support can make a significant contribution to the emotional well-being of nursing professionals (17). Also, Kandal et al. (24) emphasize the need for a supportive working environment and collaborative strategies between education and the health sector, concluding that it is essential to address unmet needs to ensure safe and quality care for patients (24).

The findings by Zhou et al. (17) and Labrague and McEnroe-Petitte (4) on work stressors for nurses reflect the complexity of the work environment. Zhou et al. (17) identified caregiving stress, role and workload stress, and peer stress and professional skills as stressors. On the other hand, Labrague and McEnroe-Petitte (4) The study analyzed the impact that the unit to which newly graduated nurses were assigned had on their perceptions of workload and stress. The findings suggest that the work context can affect the experience of stress (28). This relates to Bakker and Demerouti’s Job Demands-Resources model (2007) (7), which states that a demanding and under-resourced work environment can lead to burnout and dissatisfaction (7). For instance, Blomberg et al. (28) found high levels of stress in novice nurses, particularly in medical or surgical units, which was attributed to excessive workloads. Yeh and Yu (29) found lower levels of stress in nurses working in medical facilities, indicating that organizational interventions can reduce stress. Zhou et al. (17) also identified understaffing and multiple responsibilities as significant challenges, indicating the need to address workload at the institutional level to improve nurses’ retention. These findings underscore the importance of interventions that tackle both individual and systemic challenges related to workload and job support to promote nurse retention.

Halpin et al. (2) identified workload as a common stressor, especially during the first 12 months of employment, suggesting that novice nurses are more prone to job stress, which may influence their intention to leave. This phenomenon is exacerbated by the aging workforce, as the decline in younger workers creates a generational imbalance that increases pressure on newly graduated nurses. Tett and Meyer’s model on turnover intentions (1993) (30) reinforces this idea by pointing out that job dissatisfaction and stress contribute to the intention to leave. In addition, An et al. (21) examined the relationship between work-related stress, sleep disorders, and turnover intention among novice nurses, highlighting the importance of addressing these problems from the beginning of the career to avoid high staff turnover. This implies that stress and sleep management should be part of the initial training, as they can significantly influence the decision to change jobs (21). In this context, the study by Laschinger et al. (23) emphasizes that job dissatisfaction, influenced by unfavorable working conditions and lack of support, is one of the main causes of high turnover among novice nurses. This study also underlines that fostering positive work environments, with supportive and effective relationships, is key to improving satisfaction and reducing turnover (23).

The study by Chen et al. (6) highlights the competence of novice nurses in ethical and risk prevention areas, but points to deficiencies in team management. The positive correlation between educational level, competence in care, and intention to stay in the profession highlights the importance of continuing education for staff retention. Resilience emerges as a critical factor, particularly during the COVID-19 pandemic, where greater resilience was associated with greater intention to stay in the profession (6). A study in Jordan examined the impact of perceived stress in caring for patients with COVID-19 on nursing career decisions, finding that although many nurses chose this career voluntarily, they expressed dissatisfaction with working conditions and lack of adequate training. The pandemic has exacerbated staff shortages, generating high levels of stress and psychological trauma, particularly among inexperienced nurses. These findings indicate the need for interventions to improve nurses’ well-being, given that perception of financial income has been identified as a significant predictor of the intention to remain in the profession (25). This is related to the resilience model by Windle et al. (31), which emphasizes how coping skills can protect against the negative effects of stress in health professionals (31). These findings highlight the importance of addressing training, stress management, and resilience building to enhance the retention and well-being of novice nurses (6).

Understanding the importance of interventions that reduce work-related stress and frustration, as well as the role of nurse managers in providing support and enhancing professional competence, are key to promoting staff retention and improving quality of care (6).

The study by An et al. (21) focuses on the relationship between work-related stress, sleep disorders, and turnover intention, whereas Chen et al. (6) explore the relationship between competence, clinical stress, and intention to retain the job. Both studies highlight the need for early interventions and support to reduce turnover intention and improve retention of novice nurses. The lack of support and opportunities for skills development can demotivate novice nurses, affecting the stability of the healthcare team and leading to significant costs for healthcare organizations. Nurse leaders can promote the professional development, well-being, and engagement of novice nurses through early intervention and appropriate support, thereby contributing to the quality of care and stability of the nursing team (1).

In order to achieve well-being and satisfaction of newly graduated nurses, it is necessary to find congruence between the ‘real self’ and the ‘ideal self’, as stated in the study by Dames (22). This study highlights that discrepancies between expectations formed during university training and the reality of work may increase the risk of burnout, affecting retention in the profession. Integrating self-care and self-compassion practices into the nursing training curriculum can facilitate the adaptation of novice nurses and help them to manage work-related stress. Therefore, fostering an environment that promotes congruence and emotional management is essential for success and stability in the health care team (22).

### Limitations

Although this research has contributed to current knowledge about the stress faced by novice nurses, some limitations have been identified. Firstly, when analyzing data from different countries, great diversity in nurses’ responsibilities was found, which makes it difficult to establish an objective comparison of the results. This heterogeneity not only affects the interpretation of the data, but may also have implications for direct clinical practice, as stress management strategies may need to be adapted to specific country contexts.

Second, differences in work settings, health policies, and available resources may have the potential to introduce biases and limit the generalisability of results. It is therefore essential that future research focuses on more homogenous settings or incorporates comparative analyses.

Moreover, the inclusion of qualitative studies in the search is another limitation. This is because their exploratory nature may make it difficult to extrapolate the results.

Finally, determining when a nurse is considered a novice presented an additional challenge in assessing the included studies. This lack of clarity suggests that there is a need to standardize definitions and criteria in future research, which would not only facilitate comparison across studies, but also enrich knowledge in this field.

## Conclusion

In conclusion, this study has highlighted the various challenges faced by novice nurses in new work environments, such as workload, adaptation to the environment, professional expectations, and relationship dynamics in the team. By focusing on these aspects, a major need in the literature has been addressed, clarifying how these factors affect the intention of these professionals to remain in the profession, a topic that has been little addressed so far.

Therefore, proactive interventions, such as mentoring programs and structured training, that facilitate the adaptation of novice nurses and encourage improved teamwork are recommended. Future research would also benefit from evaluating the effectiveness of these initiatives, accounting for potential biases and the context of each setting, and from examining the role of leadership in stress reduction.

These actions seek to enhance the experience of novice nurses and also contribute to the stability of the profession, thereby ensuring quality patient care and promoting a healthier work environment. Recognizing and addressing these biases is essential to enrich understanding and strengthen the nursing practice.

## Data Availability

The original contributions presented in the study are included in the article/Supplementary material, further inquiries can be directed to the corresponding authors.
