# Supplementary material for: Stress in novice nurses in new work environments: a systematic review
Source: Front Public Health. 2024 Oct 30;12:1463751. doi: 10.3389/fpubh.2024.1463751 (PMC11557553; doi:10.3389/fpubh.2024.1463751)
Supplement: Supplementary file 1 [file Table_1.docx]

**Table S1. Scores of cross-sectional observational studies**.

| **Study** | **JBI** | **The participants and the environment are described in detail** | **Inclusion criteria are clearly defined** | **Exposure was measured in a valid and reliable way** | **The criterion used to measure the condition was objective** | **Confounding factors were identified** | **Strategies for dealing with confounding factors** | **The results were measured in a valid and reliable way** | **Appropriate statistical analysis was used** |
| --- | --- | --- | --- | --- | --- | --- | --- | --- | --- |
| (Fang et al., 2022) | 6/8 | Yes | Yes | Yes | Yes | No | No | Yes | Yes |
| (Zhang et al., 2019) | 6/8 | Yes | Yes | Yes | Yes | No | No | Yes | Yes |
| (Zhu et al., 2022) | 6/8 | Yes | Yes | Yes | Yes | No | No | Yes | Yes |
| (An et al., 2022) | 8/8 | Yes | Yes | Yes | Yes | Yes | Yes | Yes | Yes |
| (Zhou et al., 2022) | 8/8 | Yes | Yes | Yes | Yes | Yes | Yes | Yes | Yes |
| (H. M. Chen et al., 2021) | 7/8 | Yes | Yes | Yes | Yes | Yes | No | Yes | Yes |
| (Alevi, 2023) | 6/8 | Yes | Yes | Yes | Yes | No | No | Yes | Yes |

JBI: Joanna Briggs Institute tool score.

**Table S2. Scores of qualitative studies**.

| Study | JBI | Congruence between stated philosophical perspective and research methodology | Congruence between methodology and question/objectives | Congruence between the methodology and method used to collect data | Congruence between methodology and data representation and analysis | Congruence between methodology and interpretation of results | Cultural and theoretical localisation | Influence of the researcher on the sample and vice versa | Representativeness of participants | Ethical approval by an appropriate body | Relationship between conclusions and analysis or interpretation of data |
| --- | --- | --- | --- | --- | --- | --- | --- | --- | --- | --- | --- |
| (Feeg et al., 2022) | 9/10 | Yes | Yes | Yes | Yes | Yes | Yes | Yes | Yes | No | Yes |

JBI: Joanna Briggs Institute tool score.

**Table S3. Scores of cohort studies**.

| **Study** | **JBI** | **The groups were similar and were recruited from the same population** | **Exposure was measured in a similar way to classify exposed and unexposed** | **Exposure was measured in a valid and reliable way** | **Confounding factors were identified** | **Strategies for dealing with confounding factors** | **Patients did not know the objective at the start of the study** | **The results were measured in a valid and reliable way** | **Follow-up time was reported and was sufficient** | **Follow-up was completed, or if not, it was described why this was not the case** | **Strategies to address incomplete monitoring were used** | **Appropriate statistical analysis was used** |
| --- | --- | --- | --- | --- | --- | --- | --- | --- | --- | --- | --- | --- |
| (Halpin et al., 2017) | 8/11 | Yes | Yes | Yes | Yes | No | No | Yes | Yes | Yes | N/A | Yes |
| (Hoeve et al., 2020) | 8/11 | Yes | Yes | Yes | Yes | No | No | Yes | Yes | Yes | N/A | Yes |

JBI: Joanna Briggs Institute tool score; Unclear or Not Applicable: N/A

**Table S4. Scores of the systematic review.**

| **Study** | **JBI** | **The review question is clearly and explicitly formulated** | **The inclusion criteria were appropriate to the review question.** | **The search strategy was adequate** | **The sources and resources used for the search for studies were adequate** | **The assessment criteria of the studies were adequate** | **The critical appraisal was carried out by two or more reviewers independently** | **There were methods to minimise errors in data extraction** | **The methods used to combine the studies were appropriate** | **The likelihood of publication bias was assessed** | **The data reported supported policy and/or practice recommendations** | **Specific directives for further research were appropriate** |
| --- | --- | --- | --- | --- | --- | --- | --- | --- | --- | --- | --- | --- |
| (Labrague & McEnroe-Petitte, 2018) | 9/11 | Yes | Yes | Yes | Yes | Yes | No | Yes | Yes | Yes | No | Yes |

JBI: Joanna Briggs Institute tool score.

**Table S5. Scores of quasi-experimental studies.**

| **Study** | **JBI** | **It is clear which is the 'cause' and which is the 'effect' (there is no confusion about which variable comes first)** | **Participants included in some comparisons were similar** | **Participants were included in any comparison receiving similar treatment/care, other than the exposure or intervention of interest** | **There was a control group** | **There were multiple measurements of the outcome before and after the intervention/exposure** | **Follow-up was completed and, if not, differences in follow-up between the groups were adequately described and analysed** | **The results of the participants included in any comparison were measured in the same way** | **The results were reliably measured** | **An appropriate statistical analysis was used** |
| --- | --- | --- | --- | --- | --- | --- | --- | --- | --- | --- |
| (S. H. Chen et al., 2021) | 9/9 | Yes | Yes | Yes | Yes | Yes | Yes | Yes | Yes | Yes |

JBI: Joanna Briggs Institute tool score.
